# Improved filter bank common spatial pattern algorithm based on the sparrow search algorithm

**DOI:** 10.3389/fnhum.2025.1679329

**Published:** 2025-12-18

**Authors:** Yingyu Cao, Jihui Ding, Zhenxi Zhao, Yongzheng He, Moxiao Fu, Xuecheng Liu, Xiangpeng Lyv

**Affiliations:** 1 College of Mechanical Engineering, Beijing Institute of Petrochemical Technology, Beijing, China; 2 College of Mechanical Engineering, Tiangong University, Tianjin, China; 3 Xiangyu Medical Co., Ltd., Anyang, Henan, China

**Keywords:** brain computer interface, electroencephalogram, filter bank common spatial pattern, motor imagery, sparrow search algorithm

## Abstract

**Introduction:**

The application of motor imagery in human–computer interaction and rehabilitative medicine has attracted growing attention due to recent advances in brain–computer interface technologies. However, traditional EEG decoding paradigms based on fixed frequency–band segmentation often exhibit limited performance because they fail to capture individual variability in brain rhythms.

**Methods:**

This work proposes an adaptive method that integrates the sparrow search algorithm (SSA) with Filter Bank Common Spatial Pattern (FBCSP) to optimize sub–band segmentation for motor imagery EEG decoding. SSA adaptively searches for optimal sub–band boundaries, enabling individualized frequency–band selection.

**Results:**

Experiments on the BCI Competition IV 2a dataset under a cross–session evaluation protocol (training on session T, testing on session E) demonstrated that SSA–FBCSP effectively improves frequency–band adaptability. The SSA–FBCSP approach was further combined with Support Vector Machine (SVM), Linear Discriminant Analysis (LDA), and k–Nearest Neighbor (KNN) classifiers to evaluate the influence of different downstream classifiers.

**Conclusion:**

Among them, SSA–FBCSP–LDA achieved the best performance, outperforming the conventional uniform sub–band approach by 21.76% and reaching an average accuracy of 89.92%. The adaptively selected sub–bands closely matched the ERD/ERS distribution, confirming the method’s effectiveness in frequency–band optimization. Compared with recent deep–learning–based MI–EEG models, the proposed technique offers a balance of accuracy, interpretability, and computational efficiency, providing a promising direction for personalized brain–computer interface systems.

## 1 Introduction

In recent years, brain–computer interface (BCI) technology has emerged as an effective means of enabling direct communication between humans and external devices. By acquiring, processing, and interpreting electroencephalogram (EEG) signals, BCIs establish a direct link between human intention and machines while bypassing peripheral nerves and muscles (Kim et al., 2016; Wolpaw, 2007; Tonin and Millán, 2021). BCIs can be broadly categorized into invasive and non–invasive types according to their signal acquisition methods. Compared with invasive BCIs, non–invasive BCIs offer advantages such as minimal risk to users, simple setup, and convenient operation (Branco et al., 2023). They assist individuals with motor disabilities through technological aids and also facilitate communication and interaction applications (Baniqued et al., 2021; Xu et al., 2021; Brumberg et al., 2016). The most commonly adopted paradigms for non–invasive BCIs include motor imagery (MI) (Pfurtscheller and Neuper, 2001), event–related potentials (ERP) (Middendorf et al., 2000), and steady–state visual evoked potentials (SSVEP) (Farwell and Donchin, 1988).

MI has become one of the most widely applied paradigms in non–invasive BCIs. The effectiveness of MI–BCI has been demonstrated in various contexts, including stroke rehabilitation (Ming et al., 2025; Luo, 2024; Liao et al., 2023; Shen et al., 2025), prosthetic and robotic control (Qiu et al., 2018; Triana-Guzman et al., 2022), and brain signal analysis (Park et al., 2021; Shin et al., 2022; Leeuwis et al., 2021). During MI tasks, individuals are instructed to mentally rehearse specific movements, which activates corresponding cortical regions and induces event–related desynchronization (ERD) and event–related synchronization (ERS). These phenomena reflect the dynamic modulation of neural oscillatory activity in sensorimotor areas and exhibit characteristic rhythmic patterns within specific frequency bands.

To effectively capture the ERD/ERS phenomena, researchers commonly employ methods such as power spectral density (PSD) analysis (Herman et al., 2008; Xiao and Fang, 2021; Hu et al., 2024), autoregressive (AR) modeling (Lu et al., 2024), wavelet transform (Wang M. et al., 2023), and the common spatial pattern (CSP) algorithm for feature extraction from MI–EEG signals. Among these, CSP has become one of the most widely used approaches, as it enhances the discriminability of EEG features by maximizing variance differences between classes (Ramoser et al., 2000; Darvish Ghanbar et al., 2021). The integration of multi–band decomposition with CSP has further improved the characterization of EEG signals in both spatial and spectral domains. However, traditional CSP is highly sensitive to frequency–band selection and fails to adapt to inter–subject variability in rhythmic EEG patterns. Inappropriate frequency–band choices may lead to the loss of crucial ERD/ERS information. Moreover, CSP features are susceptible to non–stationarity, electrode noise, and electromyographic (EMG) artifacts, and the method tends to overfit when the data dimension is high or the sample size is limited.

To overcome the aforementioned limitations, numerous enhancement strategies have been proposed. Lemm et al. (2005) introduced the Common Spatio–Spectral Pattern (CSSP) algorithm, which combines spatial filtering with multiple first–order finite impulse response (FIR) filters. However, this method provides limited capability in accurately capturing the spectral characteristics of EEG signals and lacks an adaptive mechanism for selecting subject–specific filter bands. Dornhege et al. (2006) subsequently proposed the Common Sparse Spectral Spatial Pattern (CSSSP), which improves the representation of frequency–domain attributes by independently constructing spatial and spectral filters. Although CSSSP refines filter parameters through iterative optimization, the overall process remains computationally complex and time–consuming. Thomas et al. (2009) later developed the Filter Bank Common Spatial Pattern (FBCSP) method, which effectively exploits multi–band information to achieve superior classification performance. Building upon FBCSP, the Discriminative Filter Bank Common Spatial Pattern (DFBCSP) (Higashi and Tanaka, 2012) reduces the dependence of classification results on manually defined sub–bands. Nevertheless, its reliance on prior knowledge and predefined parameters limits its ability to adapt to individual variability in EEG frequency distributions, often leading to convergence toward local optima. In addition, regularized CSP approaches (Lotte and Guan, 2010; Lu et al., 2010) have been widely explored to improve the robustness and generalization of CSP–based algorithms. In parallel with CSP–based approaches, a number of deep–learning and transfer–learning frameworks have also been explored for MI–EEG decoding. However, these often require large–scale data and sacrifice interpretability, making CSP–based methods, particularly FBCSP, still highly competitive for small–sample and real–time BCI applications.

Among the aforementioned approaches, FBCSP is widely adopted in BCI applications because of its superior accuracy, robustness, interpretability, and scalability, and is considered the most prominent extension of CSP. FBCSP employs a fixed frequency–band partitioning strategy, dividing the 4–40 Hz range into several uniformly spaced sub–bands. Spatial pattern features are then extracted from each sub–band, thereby enhancing the representation of time–frequency–spatial characteristics. However, the conventional FBCSP method still suffers from several limitations. Its rigid frequency–band segmentation reduces algorithmic flexibility, as CSP feature extraction must be performed on all sub–bands before feature selection, and it cannot directly identify or extract features from the most informative frequency band. Moreover, when the spectral characteristics of MI signals vary considerably across individuals, a fixed sub–band division fails to account for such inter–subject variability (Tariq et al., 2019).

To further enhance the conventional FBCSP algorithm, Xiong et al. (2023) proposed an improved FBCSP framework that integrates particle swarm optimization (PSO) and adaptive spatial filtering to optimize the classification of MI–EEG signals. The PSO algorithm was employed to adjust the spatial filter parameters within the FBCSP framework, thereby enhancing classification accuracy while reducing computational redundancy. Ma et al. (2023) introduced a method termed the transformed co–space pattern (tCSP) to improve the selection of optimal frequency bands. Unlike conventional approaches, tCSP determines the most discriminative frequencies after CSP filtering, effectively avoiding feature redundancy and improving robustness.

However, many enhanced algorithms, while improving the feature extraction capability of FBCSP, have overlooked the crucial influence of sub–band division on classification performance. This omission limits further progress in decoding MI–EEG signals. In addition, the manual definition of sub–band filter groups often fails to accurately reflect the actual spectral characteristics of individual MI signals and weakens the adaptability of the algorithms. To address these challenges, this study proposes an SSA–FBCSP fusion framework based on SSA. By introducing a population–based intelligent optimization mechanism, the proposed method dynamically optimizes frequency–band parameters, refines the FBCSP filter configurations, and performs adaptive sub–band division. Through this process, the SSA–FBCSP algorithm effectively avoids convergence to local optima in sub–band partitioning, captures subject–specific EEG features more precisely, and consequently improves both adaptability and classification accuracy.

The main contributions of this paper are listed as follows:

(1)    A novel improvement of FBCSP algorithm is proposed. The method integrates SSA to achieve dynamic and adaptive configuration of the FBCSP filter bank, enabling the model to accommodate inter–individual variability in MI–EEG characteristics
(2)    The global search capability of SSA is utilized to jointly optimize the center frequency and bandwidth of each sub–band. In addition, a constraint–repair mechanism is designed to ensure the rationality of the frequency–band division
(3)    The overall effectiveness of the proposed framework is validated through ablation studies, and multiple classifiers are evaluated in terms of classification accuracy. Among them, Linear Discriminant Analysis (LDA) demonstrates the best performance and is adopted as the final classifier of SSA–FBCSP
(4)    To verify the feasibility and effectiveness of the proposed method, experiments are conducted on the public BCI Competition IV 2a dataset. The results indicate that SSA–FBCSP achieves superior classification accuracy compared with existing FBCSP–based improvement methods

The remainder of this paper is organized as follows. Section 2 introduces the research methodology, including SSA and the overall SSA–FBCSP framework. Section 3 presents the dataset and experimental settings. Section 4 reports and analyzes the experimental results and efficiency. Section 5 discusses the proposed methodology, and section 6 concludes the study and outlines future work.

## 2 Materials and methods

### 2.1 Traditional FBCSP framework

**FIGURE 1:**
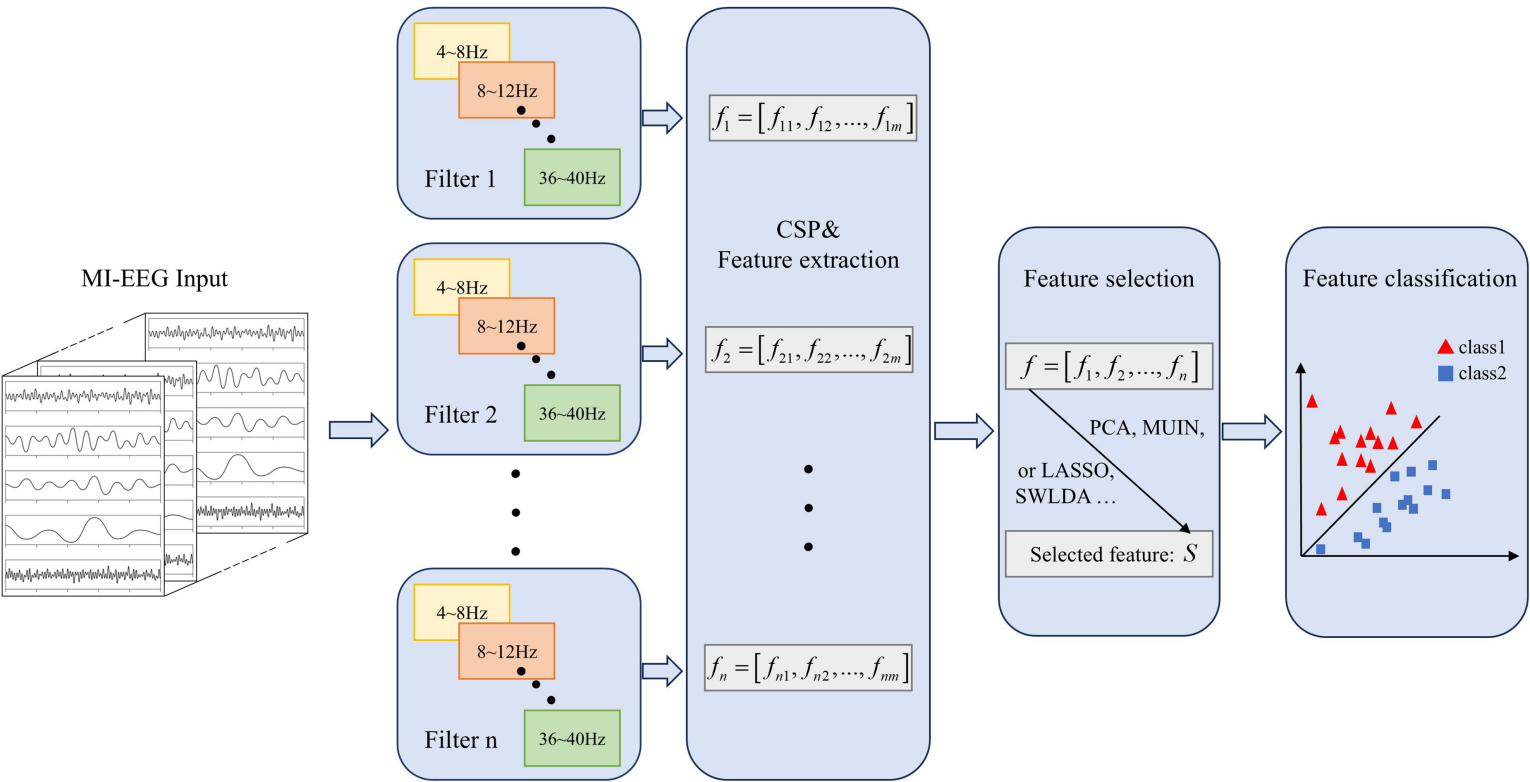
Process of FBCSP algorithm. Motor imagery EEG (MI-EEG) signals are filtered into multiple frequency sub-bands, after which the Common Spatial Pattern (CSP) algorithm is applied to extract discriminative spatial features. Features are then concatenated and reduced using PCA (Principal Component Analysis), LASSO (Least Absolute Shrinkage and Selection Operator), or SWLDA (Stepwise Linear Discriminant Analysis), before being classified into motor imagery classes.

The traditional FBCSP framework is one of the most widely used feature–extraction methods for MI–EEG classification. As illustrated in Figure 1, it decomposes multichannel EEG signals into multiple fixed sub–bands, extracts discriminative spatial patterns from each band, and concatenates the resulting features for classification. This approach enables simultaneous exploitation of spectral and spatial information and has demonstrated satisfactory performance across various BCI tasks.

In the first stage, the input MI–EEG signals are filtered through a predefined filter bank, typically covering the 4–40 Hz frequency range that contains the dominant μ (8–12 Hz) and β (18–30 Hz) rhythms. Each band–pass filter isolates one sub–band, for example 4–8 Hz, 8–12 Hz, 12–16 Hz, …, 36–40 Hz, producing n parallel signal streams which are expressed as Equation 1:


$$X_{i}\,\left(t\right),i=1,2,\ldots ,n.$$ (1)

These filtered signals preserve rhythm–specific motor–imagery information while reducing cross–band interference.

For each sub–band signal, CSP algorithm is applied to maximize the variance difference between two motor–imagery classes. Given two covariance matrices *C*_1_ and *C*_2_ corresponding to the left– and right–hand imagery trials, CSP finds a projection matrix *W* that satisfies


$$W^{T}\,\left(C_{1}+C_{2}\right)\,W=I,W^{T}\,C_{1}\,W=\Lambda ,$$ (2)

In Equation 2, Λ is a diagonal matrix containing eigenvalues that reflect the variance ratios between the two classes. The spatially filtered signal is obtained as Equation 3:


$$Z_{i}=W^{T}\,X_{i},$$ (3)

and its log–variance features form the sub–band feature vector which is expressed as Equation 4:


$$f_{i}=\left[f_{i\,1},f_{i\,2},\ldots ,f_{i\,m}\right].$$ (4)

These features capture the class–discriminative power of oscillatory patterns across electrodes.

The feature vectors extracted from all sub–bands are concatenated to form a global feature vector which is expressed as Equation 5:


$$f=\left[f_{1},f_{2},\ldots ,f_{n}\right].$$ (5)

To avoid redundancy and overfitting, dimensionality–reduction or feature–selection methods such as Principal Component Analysis (PCA), Mutual Information (MI) ranking, Least Absolute Shrinkage and Selection Operator (LASSO), or Stepwise Linear Discriminant Analysis (SWLDA) are commonly employed. The selected features Sare then fed into a linear classifier (e.g., LDA or SVM) to predict the intended motor task.

**FIGURE 2:**
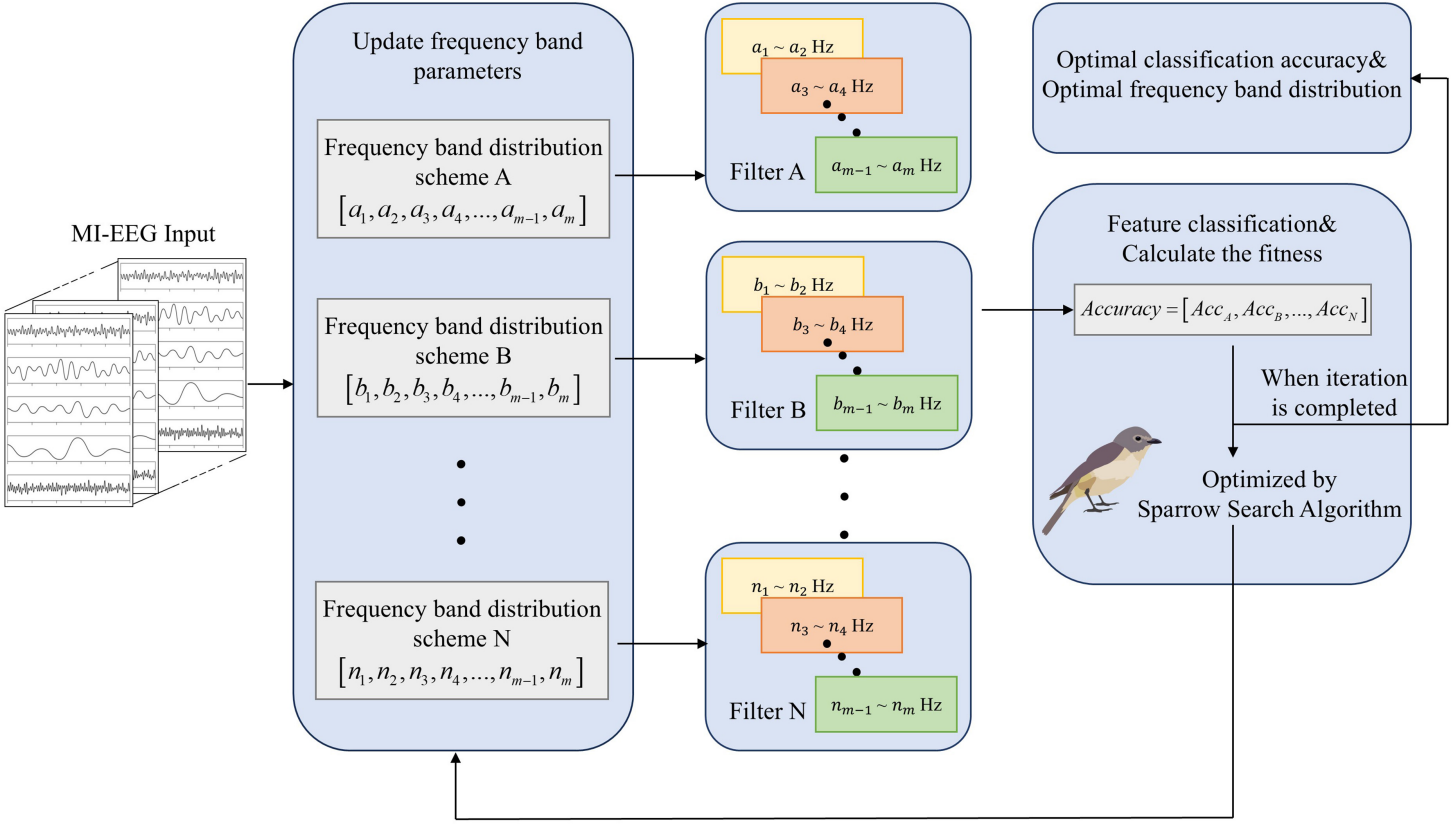
Overall framework of SSA–FBCSP algorithm. The sparrow search algorithm (SSA) generates candidate frequency-band division schemes, each defining a custom filter bank for sub-band decomposition of MI-EEG signals. For every scheme, CSP-based spatial feature extraction and classification are performed, and the resulting accuracy is used as the fitness value to update SSA parameters. This iterative process yields an individualized filter-bank structure that enhances MI classification performance.

Although the FBCSP framework effectively extracts spatio–spectral features, its performance strongly depends on the manually defined sub–band boundaries. Fixed frequency partitions cannot adapt to inter–subject variability or task–dependent spectral shifts, often leading to suboptimal feature representation. To address this limitation, an adaptive frequency–band optimization strategy based on SSA is introduced, as described in section 2.2 and illustrated in Figure 2.

### 2.2 SSA–FBCSP framework

To overcome the limitations of fixed sub–band partitioning in traditional FBCSP, an adaptive optimization framework is developed by integrating SSA with the FBCSP pipeline. As illustrated in Figure 2, the proposed SSA–FBCSP framework dynamically adjusts the central frequencies and bandwidths of the filter bank through an iterative global–search mechanism. Each sparrow individual represents a candidate sub–band distribution scheme, and the population collectively explores the optimal configuration that maximizes classification accuracy on motor–imagery EEG signals. This adaptive mechanism enables the model to better capture inter–subject variability and non–stationary oscillatory patterns in MI–EEG data.

The optimization process begins with the initialization of a population of *N* sparrows, where each individual encodes a frequency–band configuration vector *X*_*i*_ = [*x*_*i*1_, *x*_*i*2_,…, *x*_*im*_] corresponding to *m* filters within the 4–40 Hz range. At each iteration, FBCSP is executed using the candidate configuration *X*_*i*_ to extract CSP features and compute the classification accuracy *Acc*_*i*_ on the training data. As shown in Equation 6, this accuracy value serves as the fitness function guiding population evolution:


$$F\,i\,t\,n\,e\,s\,s_{i}=A\,c\,c_{i}=f\,\left(X_{i}\right).$$ (6)

The global optimum corresponds to the configuration that yields the highest mean accuracy across all folds, denoted as X* . To ensure valid sub–band boundaries, a constraint–repair mechanism is introduced to maintain non–overlapping, physiologically meaningful frequency ranges during optimization. This constraint–repair mechanism will be discussed later when describing the followers in SSA.

The optimization process continues until a predefined iteration limit *T*. After convergence, the optimal frequency–band configuration X* and its corresponding classification accuracy Acc* are obtained. The optimized sub–band boundaries are then applied to the CSP module for final feature extraction and classification, forming the complete SSA–FBCSP pipeline.

Compared with conventional FBCSP and other metaheuristic variants such as Particle Swarm Optimization (PSO) and Genetic Algorithm (GA), the proposed SSA–FBCSP framework exhibits faster convergence, stronger global exploration, and higher stability across subjects. By leveraging the adaptive role division and dynamic adjustment strategy of SSA, the algorithm effectively balances exploration and exploitation in the frequency–band search space. Consequently, SSA–FBCSP achieves superior adaptability to non–stationary EEG patterns and improves both classification accuracy and robustness, as validated in the experiments described in section 4.

### 2.3 SSA

Sparrow search algorithm is a swarm intelligence optimization technique inspired by the foraging and anti–predation behaviors of sparrows, introduced by Xue and Shen (2020). In comparison to other swarm intelligence optimization methods, SSA has advantages such as a straightforward structure, reduced parameters, and ease of implementation. SSA has been extensively applied across various research domains, including function optimization, image processing optimization, and machine learning, by incorporating the synergistic mechanism of producer–follower–watcher, which enhances global exploration capabilities and markedly increases the efficiency of local exploitation (Awadallah et al., 2023; Xue and Shen, 2024).

SSA divides the population into three functional groups—producers, followers, and watchers—each contributing distinct exploration–exploitation behaviors. Let *t* denote the iteration number and *T* the maximum iterations. The position of the *i*−*th* sparrow at iteration *t* is updated according to specific role–dependent strategies described below.

#### 2.3.1 Producers

Producers are responsible for global exploration and guiding the population toward promising search regions. They update their positions according to:


$$X_{i,j}^{\text{t}+1}=\{\begin{array}{ll} X_{i,j}^{\text{t}}\cdot \exp\left(-\frac{t}{\alpha \cdot T}\right), & if\;R_{2}<ST\\ X_{i,j}^{\text{t}}+Q\cdot L, & otherwise \end{array}$$ (7)

In Equation 7, $X_{i,j}^{t}$ denotes the *j*−*th* dimension (frequency boundary) of sparrow *i* at iteration *t*, α is a control coefficient regulating convergence speed, *Q* and *L* are components of the random perturbation term, *R*_2_ is a random number that denotes the warning value in [0,1], *ST* represents the safety threshold.

In the SSA–FBCSP technique, the producer vectors consist of many groups of band division vectors exhibiting the highest current classification accuracy, tasked with the global search for regions of high adaptability. When the random number *R*_2_ is less than *ST*, the producer vectors broaden the search area to enhance the development of the current optimal region. As the iteration count increases, the movement step of the producer vectors progressively diminishes, transitioning from a coarse search to a fine search. Conversely, when the random number *R*_2_ is greater than or equal to *ST*, the producer vectors escape the current distribution through random perturbation and proactively generate a new scheduling method to seek potentially optimal solutions, thereby avoiding local optimization traps.

#### 2.3.2 Followers

Followers exploit the information shared by producers and adjust their positions based on social cooperation. Their position update rule is expressed as:


$$X_{i,j}^{t+1}=\{\begin{array}{ll} Q\cdot \exp\left(\frac{x_{worst}^{t}-x_{i,j}^{t}}{i^{2}}\right), & if\;i<\frac{n}{2}\\ X_{best}^{t\text{+1}}+|X_{i,j}^{t}-X_{best}^{t+1}|\cdot A^{+}\cdot L, & otherwise \end{array}$$ (8)

In Equation 8, $X_{w\,o\,r\,s\,t}^{t}$ and $X_{b\,e\,s\,t}^{t+1}$ are the locations of the worst–adapted and best–adapted individuals in their respective iterations. *Q* and *L* are both randomized perturbation terms, *A* is a matrix of size 1× dim, with each element in the matrix randomly preset to −1 or 1.

In the SSA–FBCSP algorithm, the follower vectors comprise all sub–band division vectors excluding the producers, which are tasked with advancing toward the global ideal location. When *i* is less than *n*/2, it indicates that the classification accuracy achieved by the initial follower vector is inadequate, necessitating further exploration of alternative regions; conversely, when *i* is more than or equal to *n*/2, the follower progresses toward the current optimal solution.

SSA has to be improved due to the existence of band boundary constraints in the band division vector *X*_*i*_ = [*x*_*i*1_, *x*_*i*2_,…, *x*_*im*_], otherwise, the iterative process will result in erroneous bands, such as, for example, 4–2 Hz, 7–5 Hz,…40–40 Hz. The improved follower positions are as follows:


$$X_{i,j}^{t+1}=X_{i,j}^{t}+s\,t\,e\,p\cdot \left(X_{b\,e\,s\,t}^{t}-X_{i,j}^{t}\right)+Q,$$ (9)

In Equation 9, *Q* is a normally distributed perturbation and *step* is an exponential decay step with the expression:


$$s\,t\,e\,p=R\cdot \exp\left(-\frac{d_{i}}{d_{n}}\right)+\beta \,\left(1-\frac{t}{T}\right),$$ (10)

In Equation 10, *R* ∈ [0,1]is a uniform random number, *d*_*i*_ and *d*_*n*_ is the number of dimensions of the current solution vector and the maximum dimension of the solution vector, respectively, and β is the exploration weight factor.

The random number *R* guarantees that the direction of each iteration is unpredictable, preventing the follower vectors from converging on poor trajectories. The dimensional decay *step* enables the independent attenuation of the initial or final frequency of each sub–band to prevent structural inaccuracies in band division. The adaptive decay term β(1−*t*/*T*) facilitates extensive exploration of the follower vector in large increments during the initial iteration phase and allows for meticulous refinement in smaller increments during the latter iteration phase.

#### 2.3.3 Watchers

Watchers perform environmental monitoring and introduce random flight behaviors when potential danger is detected. Their update rule is defined as:


$$X_{i,j}^{t+1}=\{\begin{array}{ll} X_{best}^{t}+\beta \cdot |X_{i,j}^{t}-X_{best}^{t}|, & f_{i}\;\neq f_{b}\\ X_{best}^{t}+K\cdot \left(\frac{X_{i,j}^{t}-X_{best}^{t}}{|f_{i}-f_{w}|+\text{ε}}\right), & f_{i}\;=f_{b} \end{array}$$ (11)

In Equation 11, β is a random number that conforms to a standard normal distribution, *K* ∈ [0,1] is a uniform random number, ε is a smaller constant that prevents division by zero, *f*_*i*_ denotes the fitness value of the current individual, *f*_*b*_ and *f*_*w*_ denote the fitness values of the current global best and worst individuals.

The watcher vector in the SSA–FBCSP algorithm is chosen at random from the entire vector that includes the producers and followers. The chosen watchers vector is made to move in the direction of the global optimal solution vector if the classification accuracy it achieves is suboptimal. On the other hand, the watcher vectors are randomly reset in the event that the chosen watchers are the current optimal solution vectors, with the hope of achieving a better classification accuracy in the subsequent iteration.

In order to maintain a wide range of exploration at the beginning of the iteration and reduce the perturbation at the later convergence, the traditional formula for updating the position of watchers is improved. When *f*_*i*_≠*f*_*b*_,


$$X_{i,j}^{t+1}=X_{b\,e\,s\,t}^{t}+\beta \cdot \left|X_{i,j}^{t}-X_{b\,e\,s\,t}^{t}\right|+n\,o\,i\,s\,e,$$ (12)

$$n\,o\,i\,s\,e=\left(1-\frac{t}{T}\right)\,N\,\left(0,\sigma^{2}\right),$$ (13)

In Equations 12, 13, noise is a dynamic Gaussian noise that causes the watchers vector to escape from the local optimum and migrate to a high fitness region. *N*(0,σ^2^) is for normally distributed random numbers with mean 0 and standard deviationσ = (*f*_max_−*f*_min_). *f*_max_ and *f*_min_ correspond to the upper limit of 40 Hz and the lower limit of 4 Hz of the band range of the filter bank.

### 2.4 CSP and feature extraction

After SSA optimization, the EEG signals are passed through the Common Spatial Pattern algorithm for spatial feature extraction. As mentioned in section 2.1, CSP is a widely–used method for enhancing the discrimination between MI classes by extracting spatial filters that maximize variance differences between the two classes. This approach is applied to the optimized sub–bands obtained from SSA optimization process. The optimal frequency–band configuration, as determined by SSA, is used to filter the EEG signals, and CSP is applied to each of the sub–bands to extract the relevant spatial features.

For each optimized sub–band, the log–variance of the spatially filtered signals is computed as the feature for that sub–band. The log–variance is defined as:


$$f_{i}=\log\left(\frac{V\,a\,r\,\left(Z_{i}\right)}{V\,a\,r_{r\,e\,f}\,\left(Z_{i}\right)}\right),$$ (14)

In Equation 14, *Z*_*i*_ represents the filtered EEG signal for the *i* − −th sub–band, and *Var*_*ref*_(*Z*_*i*_) is the reference variance for normalization. The log–variance features from all sub–bands are concatenated to form the final feature vector *f*, which will be used for classification.

The extracted features are then passed to the classification stage, where various classifiers, such as Linear Discriminant Analysis (LDA), Support Vector Machines (SVM), or k–Nearest Neighbors (KNN), can be used to classify the MI signals. The classification performance of these classifiers will be evaluated through ablation experiments in section 4, where we assess their effectiveness in conjunction with the SSA–FBCSP framework.

In summary, the SSA–FBCSP method leverages SSA to optimize frequency–band boundaries, followed by CSP for spatial feature extraction. This approach enhances the representation of MI signals by adapting to individual subject–specific characteristics, improving classification accuracy and robustness. The overall performance of the SSA–FBCSP framework, including its classification performance and comparison with other methods, is discussed in section 4.

## 3 Materials

### 3.1 Dataset

The motor imagery data used in this study were obtained from the publicly available BCI Competition IV–2a dataset. This dataset includes EEG data from nine participants (labeled A01 to A09). During the experiment, participants were instructed to perform four distinct types of MI tasks: left hand, right hand, feet, and tongue.

**FIGURE 3:**
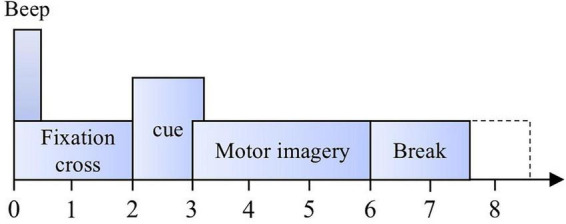
MI paradigm of BCI competition IV–2. Each trial begins with a fixation cross (0–2 s), followed by a visual cue indicating the motor imagery task (left hand, right hand, both feet, or tongue). The subject performs MI for 4 s, after which a short rest period occurs before the next trial.

Each participant performed two experiments on different dates: one for classifier training (T) and another for evaluation (E). As a result, each subject has two corresponding data files. For example, subject 1 has files A01T and A01E. Each experiment consists of six runs, each comprising 48 individual trials. In each run, the 48 MI trials comprise an equal number of samples from all four classes—12 left-hand, 12 right-hand, 12 foot, and 12 tongue imagery tasks. Although the class proportions are balanced, the presentation order is randomized. Each single trial followed the experimental paradigm illustrated in Figure 3. At the beginning of each trial, the participant entered a 0–2 s preparation phase during which a fixation cross was displayed on the screen. Starting at the 2-s mark, the motor-imagery cue appeared, instructing the participant to perform the corresponding imagery task. The cue was presented as an arrow pointing left, right, downward, or upward, indicating imagery of the left hand, right hand, feet, or tongue, respectively. The cue remained on the screen for approximately 1.25 s, and the participant continued the imagery task until the end of the 6-s interval. This was followed by a rest period of approximately 1–1.5 s before the next trial began. The experiment comprised 288 trials conducted over 6 runs, yielding a total of 5,184 experimental samples in the dataset. Trials contaminated by excessive artifacts (e.g., large-amplitude muscle or ocular artifacts) were visually inspected and rejected prior to feature extraction.

The experimental data comprised 25 channels, encompassing 22 EEG channels and 3 ocular channels. The experimental sample frequency was established at 250 Hz and subjected to bandpass filtering within the range of 0.5–100 Hz. The amplifier’s sensitivity was configured at 100 μV, and a 50 Hz notch filter was implemented to mitigate line noise.

A band-pass filter of 0.5–40 Hz was applied to the raw EEG recordings to retain the task-relevant frequency components. Because the original data contained continuous EEG signals recorded throughout the entire experiment, redundant portions had to be removed through segmentation. For each trial, the EEG from 2 to 6 s was extracted and used for subsequent feature extraction and classification. Taking one subset of the BCI-IV 2a dataset as an example, the segmented data can be represented in the format: number of sampling points *T* × number of channels *C* × number of trials *S*, where *T* = 1000, *C* = 22, *S* = 288. After segmentation, common average referencing and baseline correction were applied to obtain the final MI segments.

In accordance with the official evaluation protocol of the BCI Competition IV–2a dataset, a session-based hold-out strategy was adopted in this study. For each subject, the training session (A0xT) was used for model training, whereas the evaluation session (A0xE), recorded on a different day, served as an independent test set to assess cross-session generalization performance. This configuration ensures a realistic temporal separation between training and testing data and is widely used in prior studies on BCI IV–2a to evaluate robustness across sessions.

### 3.2 Evaluation indicators

In this paper, the SSA–FBCSP algorithm is analyzed for comprehensive performance using Accuracy (Acc) and the Kappa value.

Acc is a metric for assessing the overall predictive correctness of the algorithm and is calculated as:


$$A\,\text{cc}=\frac{T\,P+T\,N}{T\,P+F\,P+T\,N+F\,N},$$ (15)

In Equation 15, *TP*, *FP*, *TN*, *FN* represent true positives, true negatives, false positives, and false negatives, respectively.

Kappa value is a measure of the degree of consistency between the model’s prediction results and the real label, which can more objectively reflect the performance of the model in dealing with category imbalance data, and its calculation formula is:


$$K\,a\,p\,p\,a=\frac{A\,c\,c-p_{e}}{1-p_{e}},$$ (16)

In Equation 16, *p*_*e*_ represents the accuracy of classification under random conditions.

In addition to overall accuracy, Cohen’s Kappa coefficient is used in this study to provide a more reliable evaluation of classification performance under the mild class imbalance inherent in the BCI Competition IV–2a dataset. Although the dataset was designed with nominally balanced four-class MI tasks, variations in trial rejection caused by artifact contamination and subject-specific data quality inevitably introduce slight discrepancies in the number of valid trials per class. In such cases, accuracy alone may overestimate performance by failing to account for agreement occurring by chance. Furthermore, Kappa provides a chance-corrected measure of agreement that is widely recommended for multi-class MI-BCI evaluation.

Kappa corrects this limitation by quantifying the agreement between predicted and true labels after removing the contribution of random classification, thereby providing a chance-corrected and class-imbalance–aware performance measure. This makes Kappa particularly appropriate for MI-EEG evaluation, where inter-subject variability and non-stationarity often lead to uneven effective sample distributions across classes. Therefore, reporting both accuracy and Kappa ensures a more objective and robust assessment of the SSA–FBCSP algorithm.

### 3.3 Experimental setups

All experiments in this study were conducted on a workstation equipped with an Intel (R) Core (TM) i7–13700H processor (2.40 GHz), 16 GB RAM, and an NVIDIA GeForce RTX 4060 GPU. All simulations and analyses were performed in MATLAB R2023b.

To balance optimization effectiveness and computational efficiency, the parameter settings of the proposed SSA–FBCSP algorithm are summarized in Table 1. These parameters were empirically determined to ensure algorithmic stability and convergence while maintaining moderate computational cost.

**TABLE 1:** Sparrow search algorithm parameter setting.

Parameters of the algorithm	Set up
Sparrow population size	15
Number of iterations	30
Number of frequency bands	10
Safety thresholds for Producers	0.8
Percentage of Producers	0.2
Percentage of Watchers	0.3

This table lists the SSA hyperparameters, including population size, iteration count, frequency-band count, safety threshold, and the proportion of producers and watchers.

The experimental design consists of three main stages, corresponding to the evaluation, ablation, and parameter analysis presented in section 4.

The first stage focuses on evaluating the overall classification performance of the SSA–FBCSP algorithm. Its decoding accuracy was compared against four representative benchmark methods: the traditional FBCSP–SVM, and three deep learning–based models—Deep ConvNet, EEGNet–8, 2, and FBCNet. To further position SSA–FBCSP within a broader research context, comparisons were also extended to three recent deep learning–based MI decoding frameworks: an overlapping–sliding–window CNN–LSTM model, a transfer learning–based CNN–LSTM hybrid architecture, and an adaptive transfer–learning multiscale feature–fusion CNN. These comparisons demonstrate the interpretability and efficiency advantages of SSA–FBCSP relative to contemporary end–to–end deep models.

In the second stage, ablation studies were performed to analyze the internal structure of the SSA–FBCSP framework. Three commonly used classifiers—LDA, SVM, and KNN—were separately integrated into the SSA–FBCSP pipeline to determine the most suitable classification strategy. In addition, the individual contributions of the three SSA modules—producers, followers, and watchers—were examined by selectively removing each component to assess its effect on overall algorithmic stability and decoding performance.

In the third stage, parametric experiments were carried out to investigate the impact of the number of sub–bands (N) on classification accuracy. The value of N was varied within a predefined range (5–15) to identify an appropriate balance between spectral resolution and computational complexity in the filter–bank structure optimized by SSA.

Finally, to complement the accuracy-oriented evaluations, an additional efficiency analysis was conducted to assess the computational cost of the proposed SSA–FBCSP framework. This analysis focuses on both the optimization overhead incurred during SSA-based sub-band selection and the runtime characteristics of the final decoding pipeline (filtering, CSP computation, and classifier inference). Reporting these metrics provides a more comprehensive understanding of the algorithm’s practical feasibility, particularly in scenarios where real-time or near real-time MI-BCI operation is required.

## 4 Results

### 4.1 Comparison results of MI classification

To comprehensively evaluate the performance of the proposed SSA–FBCSP algorithm, comparative experiments were conducted on the BCI Competition IV–2a dataset using the same session–based hold–out protocol, where each subject’s session T was used for training and session E for testing. The SSA–FBCSP framework was compared against several representative baseline methods, including the traditional FBCSP–SVM and three widely used deep learning–based EEG decoding networks: Deep ConvNet, EEGNet–8,2, and FBCNet.

For fairness and reproducibility, the baseline results of these four methods were directly adopted from Supplementary Table 3 of the FBCNet study (Mane et al., 2021), which reports single–subject classification accuracies on the BCI Competition IV–2a dataset under the same session–based hold–out evaluation. Specifically, the performances of FBCSP–SVM (Ang et al., 2008), Deep ConvNet (Schirrmeister et al., 2017), and EEGNet (Lawhern et al., 2018) were reproduced by Mane et al. within the FBCNet framework using identical data partitions and evaluation criteria. This setting ensures that all comparison methods share a consistent experimental configuration, allowing a reliable and equitable evaluation of the proposed SSA–FBCSP algorithm against established baselines.

**FIGURE 4:**
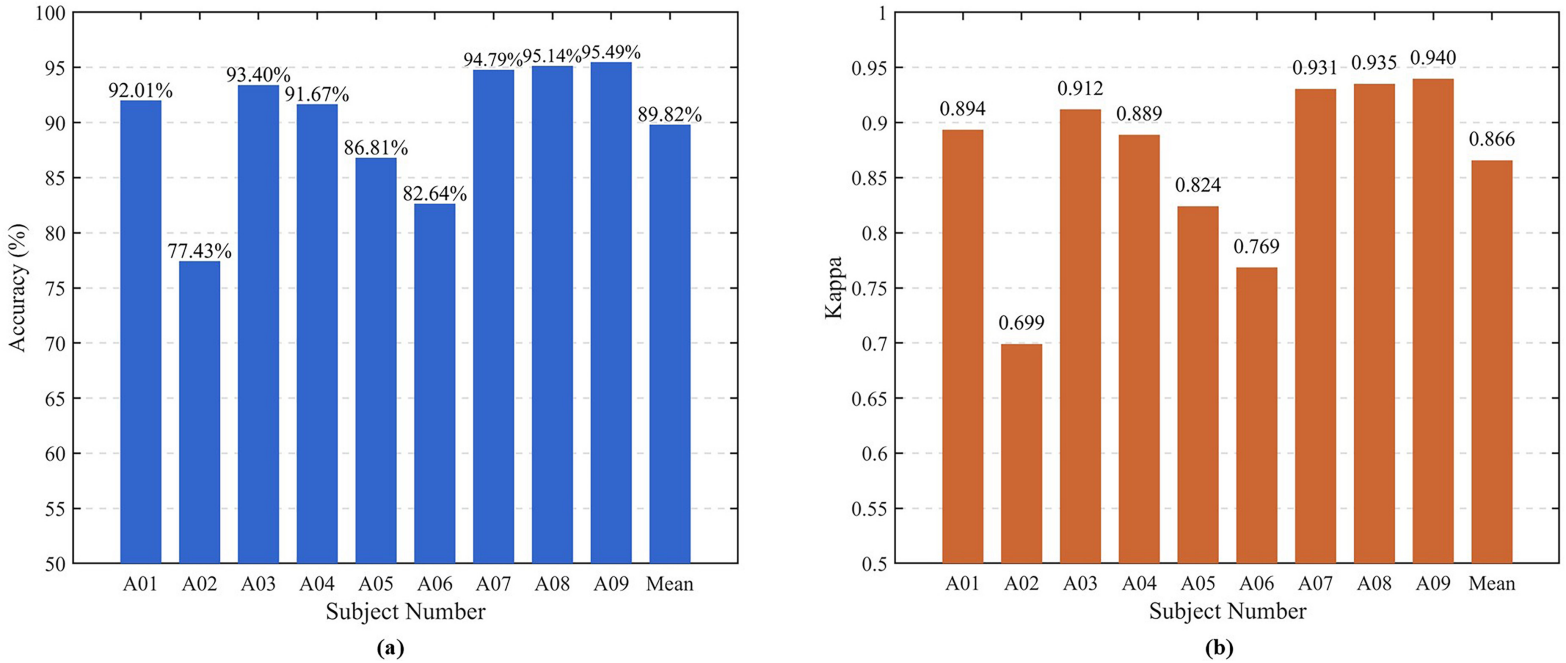
Classification accuracy and Kappa value of subjects A01-A09. **(a)** Blue bars show the classification accuracy (%) of the SSA-FBCSP algorithm for each subject. **(b)** Orange bars present the corresponding Cohen’s Kappa coefficient (×100), indicating agreement between predicted and true labels beyond chance level. SSA, sparrow search algorithm; FBCSP, Filter Bank Common Spatial Pattern.

As illustrated in Figures 4a, b, to ensure statistical reliability and mitigate the influence of random initialization, each subject’s experiment was independently repeated 10 times under the same training–testing configuration, and the average accuracy across these runs was recorded as the subject’s final performance.

As shown in Table 2, the proposed SSA–FBCSP achieved the highest classification accuracies for all nine subjects, with an average accuracy of 89.92%, which is 13.72% higher than FBCNet (76.20%) and more than 20% higher than the traditional FBCSP–SVM (68.06%). These results clearly demonstrate that adaptively optimizing the sub–band frequency distribution through SSA effectively enhances the discriminative capability of CSP–extracted spatial features. Moreover, compared with Deep ConvNet and EEGNet, SSA–FBCSP exhibits better inter–subject stability and generalization across varying EEG patterns, indicating that adaptive frequency optimization contributes to a more robust representation of MI features.

**TABLE 2:** Frequency band distribution of subjects A01-A05.

Sub–band	A01	A02	A03	A04	A05
Sub–band 1	[5.75, 10.35]	[4.00, 6.60]	[4.24, 8.39]	[4.00, 7.52]	[8.24, 9.91]
Sub–band 2	[10.35, 14.33]	[6.60, 10.45]	[8.39, 13.31]	[7.52, 10.49]	[9.91, 11.41]
Sub–band 3	[14.33, 17.23]	[10.45, 14.87]	[13.31, 16, 88]	[10.49, 12, 09]	[11.41, 13.01]
Sub–band 4	[17.23, 20.19]	[14.87, 17.92]	[16.88, 20.38]	[12.09, 14.63]	[13.01, 14.71]
Sub–band 5	[20.19, 23.27]	[17.92, 22.38]	[20.38, 22.69]	[14.63, 17.02]	[14.50, 15.60]
Sub–band 6	[23.27, 25.87]	[22.38, 26.06]	[22.69, 27.58]	[17.02, 18.80]	[14.91, 16.51]
Sub–band 7	[25.87, 28.47]	[26.06, 29.87]	[27.58, 31.70]	[18.90, 21.35]	[16.51, 17.63]
Sub–band 8	[28.47, 31.07]	[29.87, 34.03]	[31.70, 36.19]	[21.35, 23.26]	[17.69, 18.29]
Sub–band 9	[31.07, 33.67]	[34.03, 36.82]	[36.19, 39.17]	[23.66, 25.27]	[18.29, 19.89]
Sub–band 10	[32.66, 37.27]	[36.82, 40.00]	[39.17, 39.77]	[25.27, 30.86]	[20.40, 21.00]

Individualized frequency-band boundaries (Hz) optimized by SSA-FBCSP for subjects A01–A05.

The improvement achieved by SSA–FBCSP is reflected not only in the average accuracy but also in the per–subject consistency: all subjects (A01–A09) show accuracy gains of at least 7–15 percentage points relative to FBCNet. This demonstrates that SSA dynamically adjusts sub–band boundaries to match individual frequency characteristics, which is particularly beneficial for cross–subject MI decoding.

To situate the proposed SSA–FBCSP method within a broader research context, additional comparisons were made against recent deep learning–based advances in EEG motor–imagery decoding. Hwang et al. (2023) introduced an overlapping–sliding–window strategy coupled with a deep neural architecture, enhancing temporal granularity and integrating FBCSP-derived representations with LSTM-based temporal modeling. Khademi et al. (2022) proposed a transfer learning–based CNN–LSTM hybrid network that converts EEG signals into time–frequency images via continuous wavelet transform and leverages pretrained ResNet–50 and Inception–v3 models to improve generalization under limited data. Roy (2022) further developed an adaptive transfer-learning multiscale CNN (MSFFCNN) framework, emphasizing multiscale spectral extraction and subject-specific fine-tuning to boost cross-subject robustness.

These representative models reflect three major directions in state-of-the-art deep learning research—temporal sequence modeling, image-based transfer learning, and multiscale convolutional fusion—each based on end-to-end, data-driven representation learning. In contrast, SSA–FBCSP preserves the interpretability and neurophysiological grounding of traditional filter–bank CSP pipelines while introducing a global evolutionary optimization mechanism to adaptively tune the center frequency and bandwidth of each sub-band. This optimization explicitly aligns the filter bank with subject-specific μ (8–12 Hz) and β (13–30 Hz) rhythms, reinforcing the canonical ERD/ERS patterns known to dominate MI-EEG.

**FIGURE 5:**
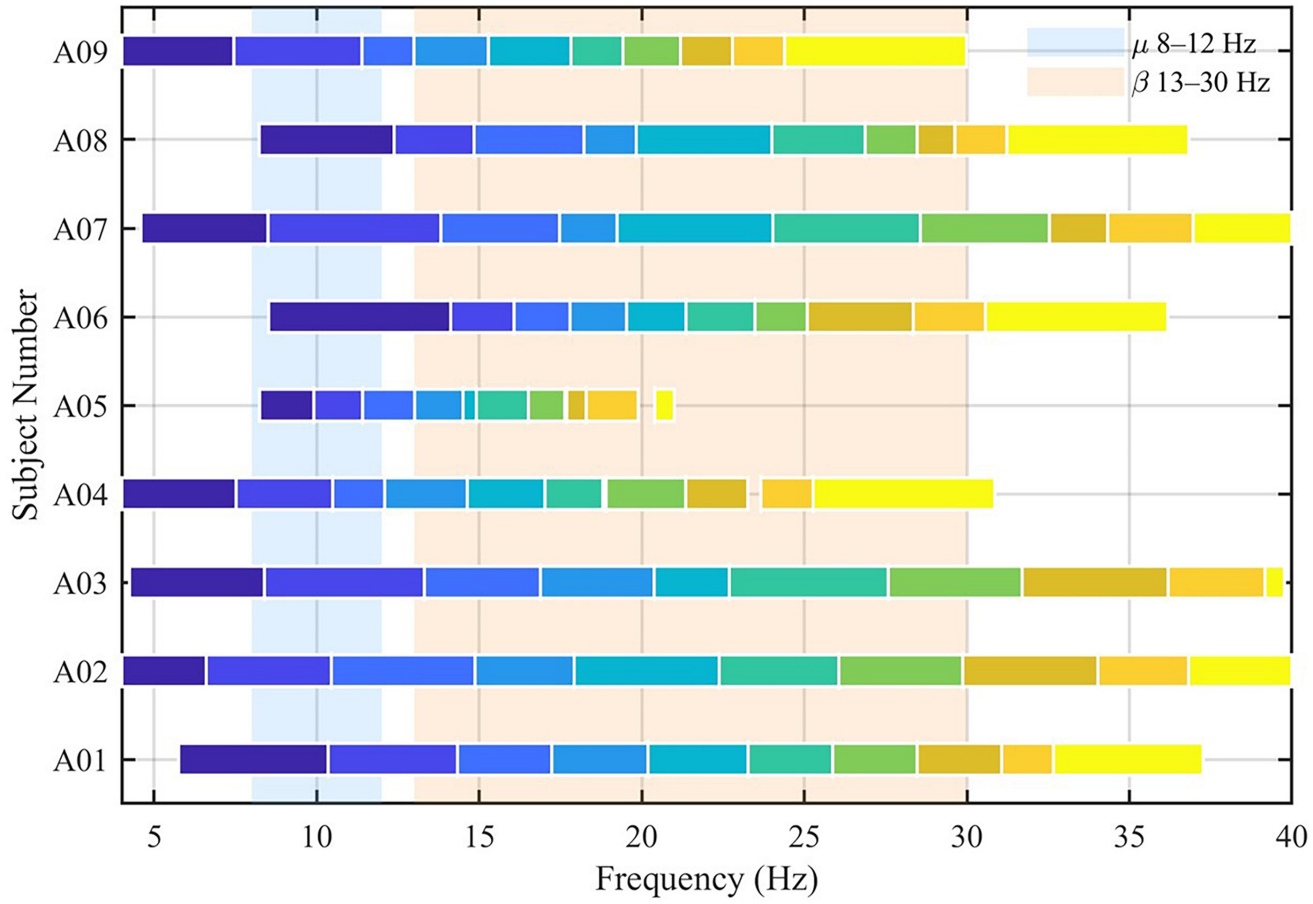
SSA–FBCSP sub–bands (single–row view) aligned with μ/ββ rhythms. Colored blocks represent individualized frequency sub-bands automatically determined by SSA during optimization. The majority of selected sub-bands concentrate within the μ (8–12 Hz) and β (13–30 Hz) rhythms associated with motor imagery–related ERD/ERS activity.

As illustrated in Figure 5, the optimized sub-bands concentrate around individualized μ /β ranges across participants, providing a clear physiological explanation for the observed performance gains. Results in Tables 2, 3 further confirm that the SSA-derived sub-band configurations capture both inter-individual spectral variability and consistent motor-related frequency centers—an aspect that purely deep-learning models typically do not expose due to their black-box nature.

**TABLE 3:** Frequency band distribution of subjects A06-A09.

Sub–band	A06	A07	A08	A09
Sub–band 1	[8.52, 14.12]	[4.59, 8.50]	[8.23, 12.38]	[4.00, 7.45]
Sub–band 2	[14.12, 16.07]	[8.50, 13.82]	[12.38, 14.84]	[7.45, 11.39]
Sub–band 3	[16.07, 17.79]	[13.82, 17.47]	[14.84, 18.22]	[11.39, 12, 99]
Sub–band 4	[17.79, 19.53]	[17.47, 19.24]	[18.22, 19.83]	[12.99, 15.28]
Sub–band 5	[19.53, 21.36]	[19.24, 24.03]	[19.83, 24.00]	[15.28, 17.82]
Sub–band 6	[21.36, 23.48]	[24.03, 28.57]	[24.00, 26.87]	[17.82, 19.42]
Sub–band 7	[23.48, 25.09]	[28.57, 32.54]	[26.87, 28.47]	[19.42, 21.19]
Sub–band 8	[25.09, 28.35]	[32.54, 34.33]	[28.47, 30.07]	[21.19, 22.79]
Sub–band 9	[28.35, 30.57]	[34.33, 36.96]	[29.63, 31.23]	[22.79, 24.39]
Sub–band 10	[30.57, 36.18]	[36.96, 40.00]	[31.23, 36.83]	[24.39, 29.99]

Individualized frequency-band boundaries (Hz) optimized by SSA-FBCSP for subjects A06–A09.

Importantly, SSA–FBCSP and deep-learning models should not be viewed as competing alternatives but rather as complementary components positioned at different stages of the signal-processing pipeline. SSA–FBCSP operates at the feature engineering and frequency optimization stage, enhancing the quality of the spectral-spatial representations fed into a classifier. Deep neural networks, by contrast, act primarily at the representation-learning or classification stage, where they can process raw signals, time–frequency maps, or CSP-derived features. Because their functional roles occur at different layers of processing, the two approaches can be combined within the same system, for example by:

(1)    using SSA–optimized sub-bands to generate enhanced CSP features as input to a deep classifier;
(2)    integrating optimized frequency bands into hybrid CNN–CSP architectures; or
(3)    embedding SSA-guided spectral priors into data-driven models to improve interpretability and reduce training burden.

Consequently, SSA–FBCSP complements rather than replaces deep learning. It provides a computationally efficient, interpretable, and data-efficient mechanism for individualized spectral optimization, while deep learning provides powerful high-level representation learning. When integrated, these approaches can form a unified pipeline that leverages both the physiological interpretability of optimized CSP features and the adaptive modeling capacity of modern neural networks, thereby offering a more robust framework for real-time or small-sample motor-imagery BCI applications.

### 4.2 Results of ablation experiments

#### 4.2.1 Selection of classifiers

In EEG signal classification research, commonly used classifiers include LDA, SVM, and the KNN. To investigate the influence of classifier choice on the performance of the proposed SSA–FBCSP framework, an ablation experiment was conducted with three experimental configurations: (1) SSA–FBCSP–LDA, (2) SSA–FBCSP–SVM, and (3) SSA–FBCSP–KNN. The classification performance of these three variants is summarized in Table 4.

**TABLE 4:** Classification results of different algorithms.

Subject no.	SSA–FBCSP–LDA	SSA–FBCSP–SVM	SSA–FBCSP–KNN
A01	92.01	88.89	86.81
A02	77.43	77.08	67.71
A03	93.40	90.28	92.01
A04	91.67	88.54	85.42
A05	86.81	76.74	69.79
A06	82.64	78.13	71.18
A07	94.79	92.36	92.01
A08	95.14	93.06	89.58
A09	95.49	91.32	92.01
Mean	89.92	86.27	82.95
Kappa	0.8,657	0.8,132	0.7,726

Classification accuracies (%) and Cohen’s Kappa of SSA-FBCSP when combined with different classifiers (LDA, SVM, KNN).

As shown in Table 4, the SSA–FBCSP model achieves the best overall performance when LDA is employed as the classification component, yielding the highest mean classification accuracy and Kappa coefficient across all subjects. Although both SVM and KNN can enhance classification accuracy for certain subjects (A01–A09), LDA proves to be more suitable for the SSA–FBCSP framework. This superiority can be attributed to its ability to maximize between–class separability while maintaining low computational complexity, which is particularly advantageous for EEG–based brain–computer interface applications.

LDA operates through a linear projection that transforms multivariate EEG features into a lower–dimensional space where class distinctions are maximized. Its simplicity and computational efficiency make it well suited for real–time processing scenarios that demand fast and stable decision–making. Consequently, the training and inference speed of SSA–FBCSP–LDA surpass those of SSA–FBCSP–SVM and SSA–FBCSP–KNN configurations, demonstrating an optimal balance between accuracy, interpretability, and computational cost.

#### 4.2.2 Removal of role modules

**FIGURE 6:**
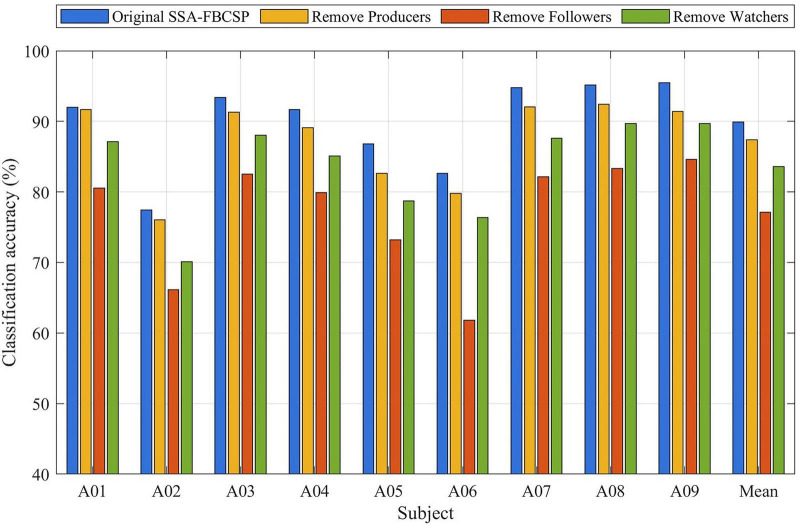
Ablation results of the SSA–FBCSP algorithm. Classification accuracy of the full SSA–FBCSP model and its ablated variants (removing producers, followers, or watchers). For each subject, four bars represent the original model and three ablation conditions. “Producers,” “followers,” and “watchers” denote the global-exploration, local-exploitation, and diversity-maintenance modules of the sparrow search algorithm (SSA), respectively.

The SSA–FBCSP framework is built upon three cooperative functional modules—producers, followers, and watchers—which together balance global exploration and local exploitation during the optimization of filter-bank boundaries. To systematically assess the contribution of each module, ablation experiments were conducted across all nine subjects (A01–A09). For each ablation condition, the model was executed ten times and the mean classification accuracy was calculated. The aggregated results are presented in Figure 6.

As shown in Figure 6, removing any single module consistently reduces decoding performance across all subjects, demonstrating that each component provides a distinctive benefit to the optimization process. The follower module exhibits the greatest impact: eliminating followers leads to accuracy reductions of approximately 11.40–25.18%, indicating their essential role in fine-grained local exploitation. Without followers, the algorithm cannot effectively refine promising frequency-band regions, resulting in substantially degraded classification performance.

Removing the watchers causes a moderate but consistent decrease in accuracy—typically within 5.28–9.47% across subjects. This confirms that watchers contribute primarily to maintaining population diversity through stochastic perturbation, thereby preventing premature convergence and improving robustness against local optima.

In contrast, removing the producers results in the smallest decline in accuracy, generally within 0.37–4.82%. Although the impact on accuracy is relatively minor, qualitative inspection shows that sub-band boundaries become less stable and occasionally irregular, reflecting the importance of producers in maintaining global search direction and guiding the optimization process.

Overall, the complete SSA–FBCSP framework achieves the highest classification accuracy for every subject, confirming that the coordinated interaction among producers, followers, and watchers yields a well-balanced global–local search mechanism. These ablation results provide strong empirical evidence that all three modules are necessary to achieve stable and effective adaptive frequency-band optimization for MI-EEG decoding.

### 4.3 Results of parametric experiments

**FIGURE 7:**
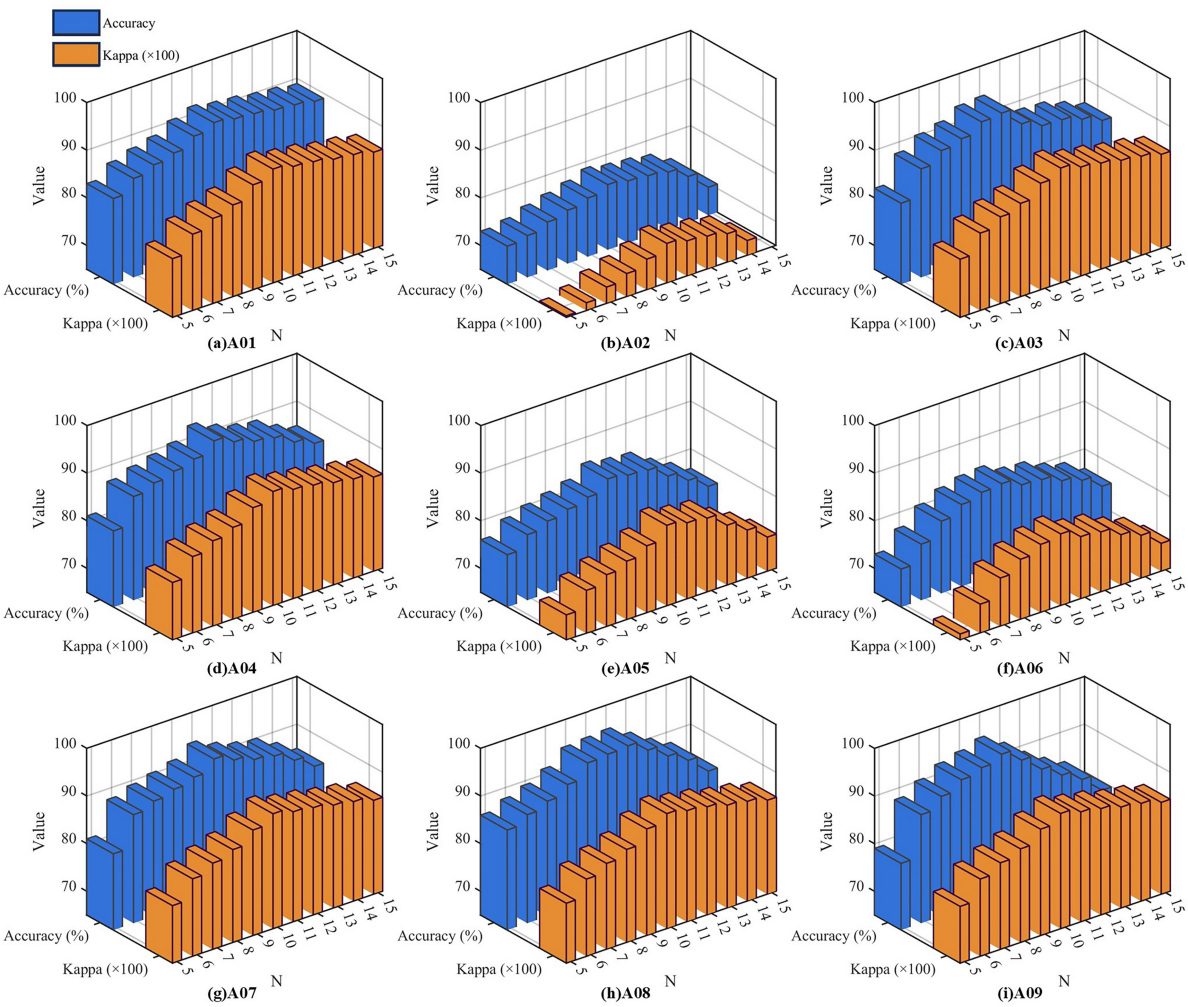
Influence of the number of sub–bands on accuracy and Kappa value. Three-dimensional visualization of the classification performance of the proposed SSA-FBCSP algorithm for nine subjects (A01–A09) under different numbers of sub-bands (*N*). Sub-figure **(a–i)** correspond to subjects A01 to A09 respectively. The blue and orange bars represent classification accuracy (%) and Kappa values (×100), respectively. Each sub-figure shows the variation of performance metrics as the number of sub-bands increases from 5 to 15.

The performance of FBCSP-based classifiers is strongly influenced by the number of sub-bands (N) in the filter bank, as this parameter governs the spectral resolution of feature extraction. To systematically evaluate its effect, N was varied from 5 to 15, and parametric experiments were conducted for all nine subjects (A01–A09) in the BCI Competition IV–2a dataset. For each subject and each sub-band configuration, the SSA-FBCSP algorithm was executed ten times under identical training–testing conditions, and the average classification accuracy and Kappa coefficient were recorded. The aggregated results are presented in Figure 7.

Across all participants, Figure 7 reveals a consistent trend in both accuracy and Kappa. Specifically, classification performance improves progressively as N increases from 5 to approximately 10, reaching a clear maximum at *N* = 10 for nearly all subjects. Beyond this point, both metrics exhibit a gradual decline. This cross-subject consistency indicates that using around 10 sub-bands offers the most favorable balance between frequency-band resolution and feature-space stability.

When the number of sub-bands is fewer than 10, the spectral partitioning becomes excessively coarse, failing to capture the detailed oscillatory characteristics associated with the motor-related μ (8–12 Hz) and β (13–30 Hz) rhythms. Although the principal ERD/ERS ranges remain included, wide sub-bands tend to merge task-irrelevant spectral components, thereby diminishing the discriminability of CSP features. Conversely, when N exceeds 10, the filters become too narrowly defined, leading to reduced data support within each band, weaker statistical robustness, and increased redundancy between adjacent bands. This finer partitioning also elevates computational complexity without providing a proportional gain in discriminative information.

Overall, the parametric analysis demonstrates that a sub-band count of approximately 10 achieves the optimal trade-off between spectral resolution, computational cost, and feature reliability. This configuration consistently yields high and stable decoding performance across subjects, underscoring its suitability for SSA-FBCSP in practical motor-imagery classification tasks.

### 4.4 Efficiency analysis

To evaluate the computational cost of the proposed SSA–FBCSP framework, we measured both the offline optimization time and the online inference latency across all nine subjects of the BCI Competition IV-2a dataset. Using a population size of 15 sparrows and 30 optimization iterations, the per-subject training and optimization time ranged from 235.1 s to 256.4 s, with a mean duration of approximately 243.9 s. This yields a total optimization time of about 36–38 min for all subjects. Such computational load is primarily attributed to repeated CSP feature extraction and classifier training inside the SSA fitness evaluations, and is acceptable for offline calibration procedures that are typically conducted only once per subject.

Once the individualized sub-band configuration was obtained, the decoding pipeline (band-pass filtering, CSP computation, and LDA classification) required 4.31 s to process an entire evaluation session, corresponding to an average per-trial inference time of 0.015 s (15 ms). This latency enables an effective real-time decoding frequency of approximately 67 Hz, which far exceeds the temporal requirements of practical MI-BCI systems, where update rates of 5–20 Hz are generally sufficient. These results indicate that while the offline SSA optimization is the computational bottleneck, the final SSA-FBCSP classifier operates with millisecond-level latency and is fully compatible with real-time BCI deployment.

## 5 Discussion

The proposed SSA–FBCSP algorithm significantly enhances the decoding of MI–EEG signals by introducing adaptive frequency–band optimization based on the SSA. Through dynamic adjustment of filter–bank sub–bands, the model effectively aligns with subject–specific μ (8–12 Hz) and β (13–30 Hz) rhythms, improving the separability of MI patterns and enhancing overall classification robustness. The results shown in Tables 2–5 indicate that SSA–FBCSP not only improves the average accuracy but also achieves more stable inter–subject performance, demonstrating the benefit of individualized spectral optimization over conventional fixed–band designs.

**TABLE 5:** Comparison of classification accuracies of different methods.

Subject no.	FBCSP–SVM	Deep ConvNet	EEGNet	FBCNet	SSA–FBCSP–LDA
A01	77.78	78.13	79.51	85.42	92.01
A02	55.56	45.14	61.11	60.42	77.43
A03	79.51	85.42	88.54	90.63	93.40
A04	63.19	67.01	71.53	76.39	91.67
A05	53.47	77.43	71.18	74.31	86.81
A06	46.88	53.13	59.03	53.82	82.64
A07	86.81	86.46	71.53	84.38	94.79
A08	81.25	78.13	80.56	79.51	95.14
A09	68.06	79.17	75.35	80.90	95.49
Mean ± Std	68.06 ± 14.11	72.22 ± 14.35	73.15 ± 9.29	76.20 ± 11.97	89.92 ± 6.32

Baseline results for FBCSP–SVM, Deep ConvNet, EEGNet–8, 2, and FBCNet are adopted from Mane et al. (2021), Supplementary Table 3, which reports per–subject accuracies under the same session–based hold–out protocol on the BCI Competition IV–2a dataset.

Similar improvements in subject–specific MI–EEG decoding through adaptive frequency optimization have also been reported in recent studies. For instance, Mousavi et al. (2022) proposed the Spectrally Adaptive Common Spatial Patterns (SACSP) approach, which adaptively learns frequency responses to maximize subject–specific discriminability. Likewise, Huang et al. (2024) demonstrated that SSA–based time–frequency segment optimization can improve EEG classification by effectively capturing non–stationary spectral dynamics. These findings are consistent with the adaptive frequency selection strategy employed by SSA–FBCSP.

When compared with recent deep–learning–based EEG decoding frameworks, SSA–FBCSP exhibits complementary characteristics rather than direct competition. Deep architectures such as Deep ConvNet, EEGNet, and FBCNet, as well as later frameworks employing overlapping–window temporal modeling, hybrid CNN–LSTM transfer learning, or multiscale adaptive fusion, emphasize end–to–end representation learning. However, they often require large datasets, heavy computational resources, and lack explicit physiological interpretability. In contrast, SSA–FBCSP retains the transparency and explainability of filter–bank CSP while achieving global optimization of frequency segmentation via SSA. Its advantage lies in being data–efficient, interpretable, and suitable for small–sample or real–time BCI applications, where individualized frequency–band optimization remains essential (Figure 5). Similar perspectives have been echoed in SSA–optimized EEG models that integrate deep belief networks for classification (Wang S. et al., 2023), further validating the efficacy of swarm–intelligence–based optimization in EEG decoding.

Despite these strengths, several limitations merit discussion. First, the current evaluation is based on an offline, session-dependent hold-out protocol and does not yet examine cross-session or cross-subject transferability.

Second, the present study performs classification using a 4-s EEG segment (2–6 s), which follows the conventional offline evaluation protocol of the BCI Competition IV-2a dataset. Although such a window length provides sufficient temporal information to capture stable μ /β rhythm desynchronization patterns, it does not reflect the latency constraints of real-time MI-BCI systems, where decision windows of approximately 1 s are commonly required. This discrepancy represents an inherent limitation of the current offline setting. To move toward online deployment, future work will investigate the performance of SSA–FBCSP under shorter sliding windows and continuous data-stream conditions, and evaluate how the learned individualized sub-band configuration can be incorporated into real-time decoding pipelines without compromising responsiveness.

Third, although the proposed framework substantially improves classification accuracy, the SSA-based optimization stage introduces additional computational overhead. This cost is manageable in offline analyses but has implications for real-time BCI deployment. Techniques such as adaptive parameter scheduling could help reduce optimization costs in future implementations.

Finally, the reliance on CSP implies sensitivity to noise and inter-trial non-stationarity. These issues could be mitigated by incorporating artifact-resistant preprocessing pipelines, Riemannian spatial filtering, or hybridized frameworks that integrate interpretable CSP-based features with lightweight deep-learning modules for improved robustness (Kumar et al., 2024).

In summary, SSA–FBCSP bridges the gap between interpretable filter-bank spatial pattern analysis and modern adaptive optimization strategies. By enabling individualized spectral tuning and retaining high computational efficiency, it provides a physiologically grounded and practical solution for both offline EEG research and future real-time MI-BCI applications. Future work will focus on reducing computational cost, exploring adaptive parameter scheduling for SSA, and extending the framework toward cross–session generalization and noise–resilient preprocessing.

## 6 Conclusion

This study proposed an adaptive motor–imagery EEG classification framework, SSA–FBCSP, which integrates SSA with the FBCSP method. By dynamically optimizing the center frequencies and bandwidths of sub–bands, SSA–FBCSP achieves subject–specific alignment with μ (8–12 Hz) and β (13–30 Hz) rhythms, thereby enhancing the discriminability and stability of extracted spatial features. Experimental results on the BCI Competition IV–2a dataset confirmed substantial improvements in classification accuracy and inter–subject robustness compared with both traditional FBCSP and recent deep–learning–based approaches. Beyond achieving high accuracy, SSA–FBCSP preserves the interpretability and computational efficiency of traditional CSP–based methods, offering a data–efficient and explainable alternative for small–sample or real–time BCI applications. Given its individualized frequency–band optimization and strong decoding performance, the proposed SSA–FBCSP algorithm provides a promising foundation for next–generation brain–computer interfaces, particularly in medical–rehabilitation applications such as stroke recovery and assistive communication.

## Data Availability

Publicly available datasets were analyzed in this study. This data can be found at: https://www.bbci.de/competition/iv/#download.
